# Dr. George S. Trevor

**Published:** 1916-05

**Authors:** 


**Dr. George S. Trevor** (not listed in Polk) died at his home in Detroit, April 4, aged 77. He was in the oil business but formerly practiced medicine in Lockport.

